# Making guidelines, research and scientific papers as simple as possible

**DOI:** 10.1080/13814788.2019.1635368

**Published:** 2019-07-10

**Authors:** Carl Llor

**Affiliations:** Via Roma Primary Healthcare Centre, Barcelona, Spain

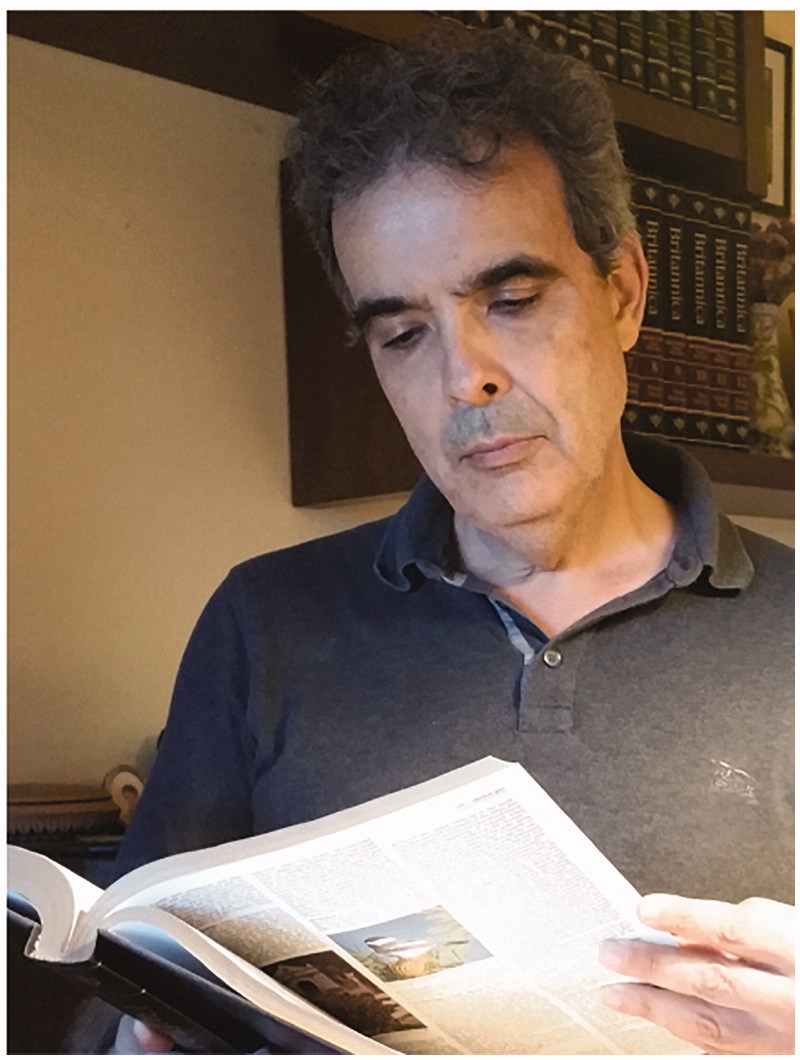

Life is really simple, but we insist on making it complicated [1]. *—Confucius*

We live in a world in which we are constantly busy, and there are seemingly endless numbers of options for everything [2]. At each turn, life seems to present numerous complications. It becomes hard to resist the allure of doing more things and trying to solve a myriad of problems. We believe that life is much more complicated than it really is. Conventional wisdom tells us that greater choice is for the greater good, but is this correct?

Primary care can benefit the population by improving overall long-term patient care and health with preventive and educational measures, appropriate and focused provision of care, and evidence-based management of acute and chronic physical, mental and social health issues. This means that primary care involves the broadest scope of healthcare. Consequently, a general practitioner (GP) must possess a wide breadth of knowledge in many areas. Not everyone knows or even needs to know the details of all the medical conditions we are handling. Also, not everyone can afford the time or have sufficient skills to be able to cope with lengthy medical rigmarole.

In the same way that we often, consciously or unconsciously, covet a more straightforward approach to life, we unintentionally try to embrace simplicity as primary care professionals. This principle, also called parsimony, is the idea that simpler explanations of observations are preferred to those that are more convoluted—conventionally referred to as Occam’s razor. As GPs we are obliged to reduce unnecessary and inappropriate medical care, following the ‘Less is More’ policy and make things as simple as we can. We must communicate with our patients simply and straightforwardly. The acronym KISS or ‘Keep it simple, stupid’ is a valuable piece of advice when communicating with patients. No matter how complicated the situation is, we must try to ask ourselves, ‘Is this as fair, clear, reliable and to the point as required?’

Guidelines are meant to simplify the management of diseases and increase GP adherence [3]. Recent years have seen the development of a wide array of evidence-based guidelines for clinical practice. High-quality healthcare has various spheres but its essence is the need to ensure that care is effective. It is, therefore, important that patients receive the most convenient care every time that they are treated. Studies indicate a clear association between lack of adherence and worse clinical outcomes. For example, lack of adherence to guidelines has found to increase hospital admissions, mortality rate, quality of life and loss of productivity in patients with chronic obstructive pulmonary disease (COPD) [4]. Common themes arising in qualitative studies on lack of GP adherence to guidelines are that complexity makes it difficult to follow or clinicians feel it is not relevant to their situation. Strategies to improve guideline usage tend to focus on dissemination and education. These approaches, however, do not address some of the more complex individual and systemic factors that influence whether a guideline is used or not in clinical practice. To consider approaches to improving GP adherence to guidelines, understanding barriers to guideline adherence is essential. Despite many clear and straightforward guidelines, an increasing number are hard to follow, confusing, and sometimes lack agreement. Consider, for example, the different guidelines available for such common conditions as the requirement for anticoagulation, management of sore throat or treatment of COPD. Grol et al. found that the implementation of guidelines that were precisely and clearly defined by clinicians was much higher compared with vague and non-specific guidelines (67% vs 36% of the occasions, respectively) [5]. Not only GPs prefer simpler guidelines. Michie et al. carried out a clinical trial in which mental health service users received either the original text of the NICE public guidelines for the management of schizophrenia or a behaviourally specified text with the same content, but in a simpler format [6]. The latter led to stronger intentions to implement the guidelines, more positive attitudes towards them, and greater perceived behavioural control overusing them. As Schwartz argues in his 2004 book *The Paradox of Choice*, providing infinite choice is paralyzing and exhausts the human psyche [7]. Similarly, a complicated guideline or algorithm with multiple choices can be frustrating. Guidelines must assist physicians with the best treatment in a given situation and avoid considering numerous possibilities, especially when guidelines for the same condition are different. Too much choice undermines happiness; too many guidelines, which are not always homogeneous, undermine confidence and adherence.

Simplicity should also be present in research. Primary care undoubtedly needs more research and research needs more primary care. Both our research question and the methodology used to answer this question should be as straightforward as possible. As Einstein put it, our theories should be ‘as simple as possible but no simpler.’ As a member of the Editorial Board of the *European Journal of General Practice*, I can clearly state that we sometimes receive complex research studies or papers that are complicated and dull to read. Complex studies also undermine the clinicians’ confidence. Take, for example, the composite outcomes used in many papers in cardiovascular medicine. As Cordoba et al., mention in their landmark paper, a drug that leads to a large reduction in a composite outcome of ‘death and chest pain;’ their finding could mean that the drug resulted in fewer deaths and less chest pain but it is possible that the composite is driven entirely by a reduction in chest pain with no change or even an increase in death [8].

When writing a paper we should be as simple as possible. As with all other types of writing, complicated articles also have to be written with the reader profile in mind. One needs to focus on a particular topic and write strictly within the scope of such confines, and one has to be realistic when setting these boundaries. An excellent article is not one that is more complicated; a good article makes the reader think and can create discussion. Like a brilliant speaker, a speech should reflect the level of the audience and the audience has to be able to understand what the speaker says because complex speeches can easily become dry and dull if not handled carefully. Presenting evidence and recommendations in a clear, concise, accessible, flexible and simple format facilitates the retrieval and assimilation of specific information. Therefore, simple language, unambiguity, relevancy of content, and clarity of structure and logic in the document are crucial to ensure that the information flows and that the subject matter is easily understood by the people who need to understand it and to make the right decisions.

Not everyone considers the same degree of simplicity a theory, a guideline, an algorithm, a piece of research, etc. Something simple in one approach may seem complex in another, suggesting that simplicity lies in the eye of the beholder. Anyone can make things bigger and more complex. However, being as simple as possible is the wisest, albeit not necessarily the easiest, choice for being successful. It takes talent and courage to move this way. Keeping things simple should be mandatory in primary care.
